# *Deinostigma fasciculatum*, a new species of Gesneriaceae in Yunnan, China

**DOI:** 10.3897/phytokeys.157.32683

**Published:** 2020-08-26

**Authors:** Yu-Min Shui, Jian-Yong Wu, Zhi-Yong Yu, Shi-Wei Guo, Li Chen, Fang Wen, Wen-Hong Chen

**Affiliations:** 1 CAS Key Laboratory for Plant Diversity and Biogeography of East Asia, Kunming Institute of Botany, Chinese Academy of Sciences, 132 Lanhei Road, Kunming 650201, Yunnan, China; 2 Nanjing Institute of Environmental Sciences, Ministry of Ecology and Environment, Nanjing 210042, China; 3 Jinping Management Bureau, Fenshuiling National Nature Reserve, Jinping 661500, Yunnan, China; 4 Guangxi Institute of Botany, Guangxi Zhuang Autonomous Region and Chinese Academy of Sciences, Guilin 541006, Guangxi, China; 5 University of the Chinese Academy of Sciences, Beijing 100049, China; 6 Karst Conservation Initiative of Yunnan, Kunming 650201, Yunnan, China; 7 School of Life Sciences, Yunnan University, Kunming, 650091, Yunnan, China

**Keywords:** *Deinostigma cicatricosum*, *Deinostigma cyrtocarpum*, new species, Sino-Vietnamese border, Yunnan

## Abstract

A new species of *Deinostigma* (Gesneriaceae) from Yunnan, China, *Deinostigma fasciculatum* W.H.Chen & Y.M.Shui, sp. nov., has been discovered and described. In the genus, the new species is similar to *D. cicatricosum* (W.T. Wang) D.J. Middleton & Mich. Möller and *D. cyrtocarpum* (D. Fang & L. Zeng) Mich. Möller & H.J. Atkins in dark purple flowers and falcate fruit, but differs from them mainly in the inflorescences with fasciculate flowers, calyx lobes (reflexed, narrowly lanceolate and 1.2–1.3 cm long), corolla tubes (sharply contracted below middle and white outside and below throat). The above three species grow nearby non-limestone wet cliffs and geographically isolated with different distributions (the new species in Southeast Yunnan, *D. cicatricosum* in Eastern Guangxi and *D. cyrtocarpum* in Southern Guangxi and Guangdong, China).

## Introduction

The genus *Deinostigma* W.T. Wang & Z.Y. Li (Gesneriaceae) was established in 1992, based on the type species *D. poilanei* (Pellegr.) W.T. Wang & Z.Y. Li which was transferred from *Hemiboea* Clarke, from South of Vietnam (Wang and Li 1992). Möller et al. (2016) enlarged this genus to 7 species, including some species in *Deinostigma* and previously in *Primulina* in South of China and Vietnam, based on molecular (ITS and *trn*L-F regions), morphological and cytological characters. Five Vietnamese species are in the genus and all distributed in Central Vietnam and South Vietnam, far from the border with China, viz. *Deinostigma cycnostylum* (B.L.Burtt) D.J.Middleton & H.J.Atkins, *D. eberhardtii* (Pellegr.) D.J.Middleton & H.J.Atkins, *D. minutihamatum* (D.Wood) D.J.Middleton & H.J.Atkins, *D. poilanei* (Pellegr.) W.T.Wang & Z.Y.Li, *D. tamiana* (B.L.Burtt) D.J.Middleton & H.J.Atkins. Up to now, two Chinese species, viz. *Deinostigma cyrtocarpum* (D. Fang & L. Zeng) Mich. Möller & H.J. Atkins and *D. cicatricosum* (W.T.Wang) D.J. Middleton & Mich. Möller are recognised as members of the genus (Wang 1981; Fang et al. 1993). Although *D. cicatricosum*, formerly *Chirita cicatricosa* W.T. Wang, was regarded as a synonym of *Chirita minutihamata* D. Wood from Vietnam (Wang et al. 1990, 1998; Li and Wang 2005), Möller et al. (2016) and Wen et al. (2019) still recognised *D. cicatricosum* in the genus.

Previous orthography of species epithets in *Deinostigma* has used the feminine ending (i.e., D. "*cycnostyla*", see Möller et al. 2016). The generic name *Deinostigma* is neuter however, and so all epithets have been corrected here (e.g., to *Deinostigma cycnostylum*) to comply with Article 62.2(c) of the ICN.

**Figure 1.**
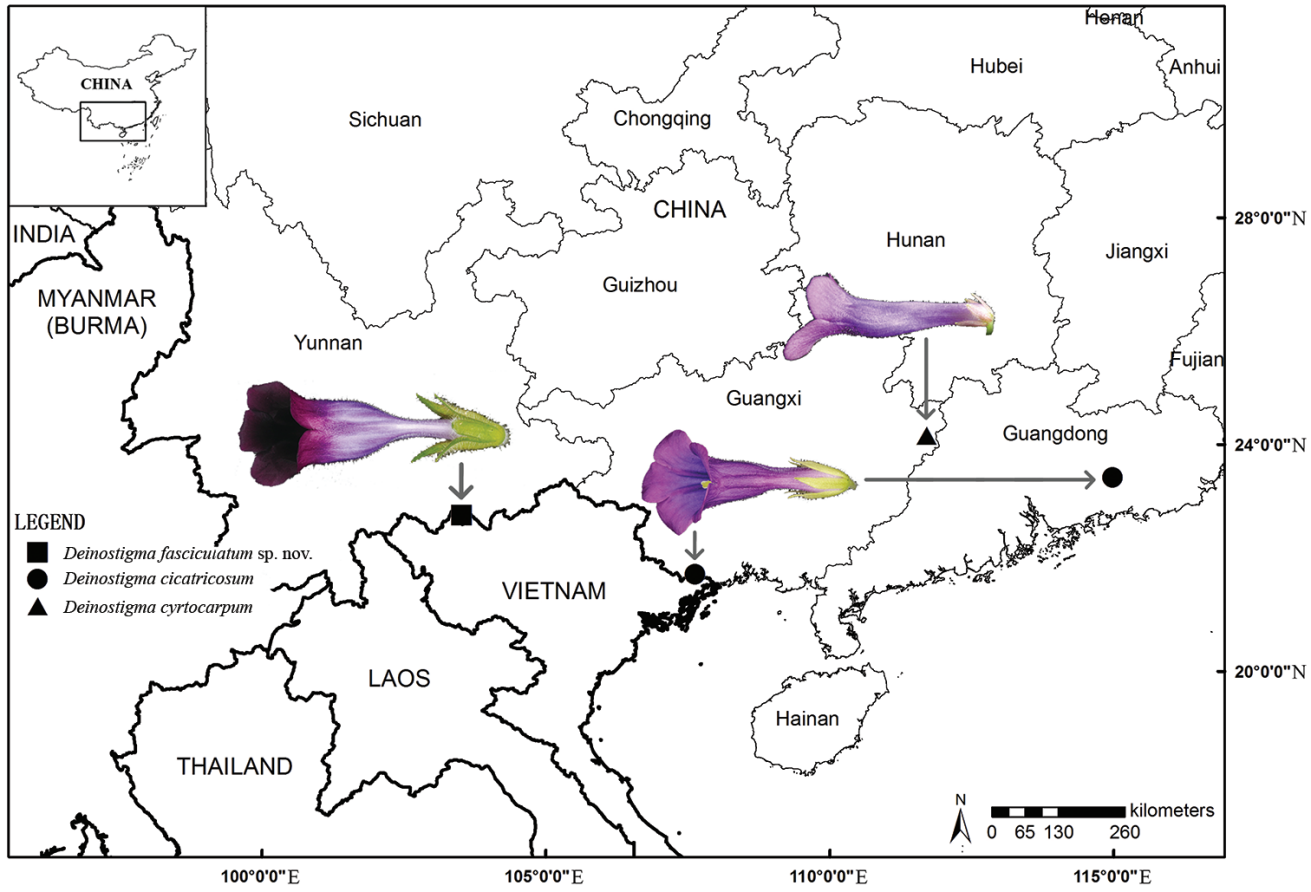
The distribution of *Deinostigma fasciculatum* W.H.Chen & Y.M.Shui, sp. nov. (■), with *D. cicatricosum* (W.T.Wang) D.J.Middleton & Mich.Möller (●) and *D. cyrtocarpum* (D.Fang & L.Zeng) Mich.Möller & H.J.Atkins (▲).

**Figure 2.**
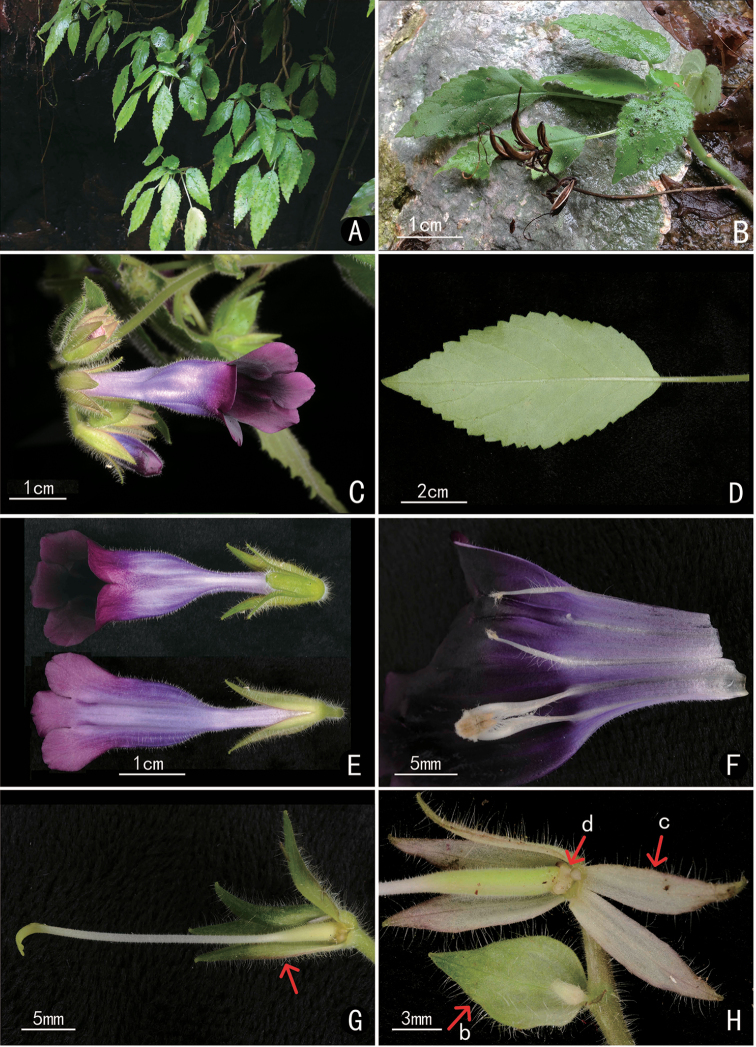
*Deinostigma fasciculatum* W.H.Chen & Y.M.Shui, sp. nov. **A** habit **B** mature fruits **C** frontal view of flower **D** leaf abaxial side **E** top and back view of flowers **F** top view of opened corolla showing the interior surface of corolla tube, stamens and staminodes **G** pistil and calyx, arrow showing the calyx **H** ovary, calyx and bract. (b = bract, c = calyx, d = disc).

**Figure 3.**
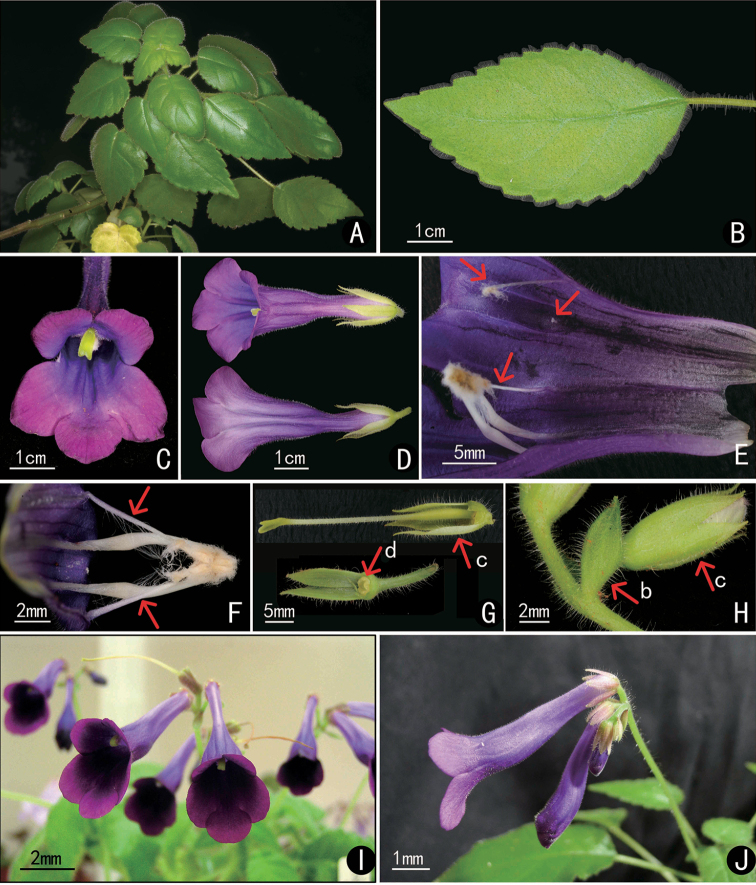
Photographs of *Deinostigma cicatricosum* (W.T. Wang) D.J. Middleton & Mich. Möller (**A–H**) and *D. cyrtocarpum* (D. Fang & L. Zeng) Mich. Möller & H.J. Atkins (I & J) **A** habit **B** adaxial surface of leaf **C** frontal view of flower **D** top view of flower **E** opened corolla showing the interior surface of corolla tube, stamens and staminodes, arrows showing the staminodes **F** stamens and staminodes, arrows showing the staminodes **G** pistil and calyx **H** bract and young flower **I** inflorescence of *D. cyrtocarpum***J** lateral view of inflorescence (b = bract, c = calyx, d = disc).

After the surveys in the Sino-Vietnamese border (Fig. 1), a new species of *Deinostigma* from Jinping county, Yunnan province, China, has been confirmed and described. Careful examination of the type specimens and related publications reveals that the new species is more similar to *D. cicatricosum* (W.T. Wang) D.J. Middleton & Mich. Möller and *D. cyrtocarpum* (D. Fang & L. Zeng) Mich. Möller & H.J. Atkins than the other five Vietnamese species in fruit morphology (Wang et al. 1998; Wei et al. 2010; Möller et al. 2016; Wen et al. 2019). Although the above three Chinese species are similar to each other in habit and falcate fruit, the floral morphology and geographical distribution provide evidence to identify them respectively (Figs 1–3, Table 1).

**Table 1.** Morphological comparison between *Deinostigma fasciculatum* sp. nov., *D. cicatricosum* and *D. cyrtocarpum* in China.

Characters	*Deinostigma fasciculatum*	*D. cicatricosum*	*D. cyrtocarpum*
**Leaf base**	often slightly peltate	seldom peltate	often slightly peltate
**Inflorescences**	with fasciculate flowers	with remote flowers	with remote flowers
**Calyx lobes**	narrowly lanceolate, 12–13 × ca. 2 mm, inside sparsely glandular villous	narrowly oblong, 8–10 × 1.8–2.5 mm, inside nearly glabrous	narrowly oblong, 5–8 × 1–2 mm, inside nearly glabrous
**Calyx margin**	margin reflexed	margin compacted	margin compacted
**Corolla tube**	outside white, sharply contracted below throat	outside purple, slightly contracted	outside purple, gradually contracted
**Capsule**	narrowly oblong	linear	narrowly oblong
**Locality**	Southeast Yunnan, China	Southern Guangxi and Guangdong, China	Eastern Guangxi, China
**Altitude**	500–850 m	300–737 m	100–200 m

## Materials and method

We observed the morphology of the species and took photographs of the habitat and macro-morphological characters, both during the fieldwork in Jinping County, South-eastern Yunnan, China and at Kunming Botanical Garden. We also examined the specimens of *Deinostigma* in the herbaria (E, KUN, P & PE). All micro-morphological characters were observed and photographed with a Leica S8 APO stereomicroscope (Shanghai, China) and a Nikon D700 microscope camera (Tokyo, Japan).

## Taxonomy

### Deinostigma fasciculatum

Taxon classificationPlantaeLamialesGesneriaceae

W.H.Chen & Y.M.Shui sp. nov.

80FFF8FA-5124-5845-928C-8FBDCB4F76CE

urn:lsid:ipni.org:names:77211196-1

Figure 2

#### Type.

China. Yunnan province, Jinping County, Ma-an-di town, 22°58'33"N, 104°50'32"E, 11 August 2018, collected from the living plants at Kunming Botanical Garden, *Y.M. Shui & S.W. Guo B2018-493* (holotype, KUN!).

#### Diagnosis.

The new species is similar to *D. cicatricosum* and *D. cyrtocarpum* in dark purple flowers and falcate fruit, but differs from the latter two species in the inflorescences with fasciculate flowers (*vs.* with remote flowers), calyx lobes reflexed (*vs.* compacted), corolla tubes white outside and below throat (*vs.* purple) (Figs 2C, 3). The new species differs from *D. cicatricosum* in calyx lobes narrowly lanceolate (*vs.* narrowly oblong) and 1.2–1.3 cm long (*vs.* 0.8–1.0 cm), corolla tube sharply contracted below middle (*vs.* slightly contracted), capsule narrowly oblong (*vs.* linear) 2–2.5 cm long (*vs.* 3–4 cm long). It differs from *D. cyrtocarpum* in calyx lobes 1.2–1.3 cm long (*vs.* 0.5–0.8 cm long), corolla tube sharply contracted (*vs.* gradually contracted).

#### Herbs perennial.

Stems pendulous, 30–60 cm long, densely glandular villous. Leaves alternate near stem apex; petiole 2–3.5 cm long, densely glandular villous; leaf blade herbaceous, ovate, elliptic or cordate, 3–9 × 2.5–4 cm, base oblique, often slightly peltate, cuneate, cordate or round, apex acuminate, margin serrate, adaxially densely glandular villous, abaxially densely glandular villous; venation penninerved, lateral veins 3–5 on each side of mid-rib. Cymes axillary near stem apex, fasciculate; peduncle 1.5–11.5 cm long, densely glandular villous; bracts 2, ovate, caducous, 0.8–1.2 × ca. 0.6 cm, adaxially sparsely glandular villous, abaxially densely glandular villous; bracteoles 2, lanceolate, caducous, ca. 0.6 × 0.2 cm, adaxially sparsely glandular villous, abaxially densely glandular villous; pedicel ca. 0.5 cm long, densely glandular villous. Calyx 5-parted to the base, segments lanceolate, 1.2–1.3 × ca. 0.2 cm, apex acute, margin entire, outside densely glandular villous, inside sparsely glandular villous. Corolla funnelform, zygomorphic, 3.5–4 cm long, ca. 1 cm wide at the throat, outside dark purple, densely glandular pubescent, inside dark purple, glabrous; tube ca. 2.5 cm long; limb 2-lipped, adaxial lip 2-lobed, lobes semi-circular, ca. 0.8 cm long, 0.5 cm in diam. at base; abaxial lip ca. 1.5 cm long, 3-lobed, middle lobes orbicular, ca. 0.5 × 0.5 cm, lateral lobes orbicular, ca. 0.5 × 0.6 cm. Stamens 2, adnate to corolla tube ca. 1.5 cm from base, coherent; anthers densely villous; filaments densely villous, ca. 1.2 cm long; staminode 3, lateral 2, villous, slightly coherent with the anthers, adnate to corolla tube ca. 1.5 cm from base, ca. 0.8 cm long; middle 1, adnate to corolla tube ca. 1.5 cm from base, ca. 1 mm long. Disc ring-like, ca. 1 mm high. Pistil ca. 3.5 cm long; ovary linear, densely glandular pubescent, ca. 0.8 cm long; style linear, ca. 2.7 cm long; stigmas obtrapeziform, emarginate. Capsule obliquely narrowly oblong, 2–2.5 cm long, curved.

#### Phenology.

Flowering is from May to August and fruiting from July to September.

#### Etymology.

The name refers to the flowers, which are fasciculate on inflorescences of the new species.

#### Vernacular name.

Cù Huā Qí Zhù Jù Tái (Chinese pronunciation); 簇花奇柱苣苔 (Chinese name).

#### Distribution and habitat.

The new species only grows on the wet cliff in the valley and only occurs at the type locality, Jinping County, Yunnan province, China.

#### Additional examined specimens.

China. Yunnan province: Jinping county, Ma-an-di town, 22°58'33"N, 104°50'32"E, in valleys, alt. 500 m a.s.l., with fruits, 22 January 2016, *Y.M. Shui & W.H. Chen B2016-084* (KUN!). The same county, Ma-an-di town, Maguaitang village, on wet cliff, alt. 520–850 m a.s.l., with buds, 1 May 2019, *Z.Y. Yu B2019-001* (KUN!).

#### Conservation state.

The new species has been only observed from the type locality in the nature reserve, with ca. 30, 000 m^2^ area (300 m × 100 m) and ca. 160 mature individuals on the cliff. The type locality is located in a deep valley with a small power station. Occasionally, local people go there to camp. Additionally, due to the building of a road, some of the slopes may become unstable and fall, resulting in some individuals being destroyed in the future. So, we hereby assessed the new species as “Critically Endangered (CR)” (C2+a+ii or B2+b+iii). (IUCN 2012, 2017).

#### Note.

*Deinostigma cyrtocarpum* is easily distinguished from *D. cicatricosum* and *D. fasciculatum* by its short calyx (Figs 1, 3J). Secondly, in *D. cicatricosum* and *D. fasciculatum*, corolla tubes are obviously contracted at the middle. As to the L/U ratio (width of lower part/width of upper part of corolla tube), the L/U ratio of *D. cicatricosum* is about 1/2.5 and lightly contracted, while the L/U ratio of *D. fasciculatum* is about 1/5 and sharply contracted (Figs 2, 3). Besides, after the careful examination of the type specimens, *Deinostigma minutihamatum* is distributed in Central Vietnam with 2300 m elevation and characterised by almost straight capsules instead of falcate capsules and so considerably different from the Chinese species of the genus with falcate capsules (Fig. 2B; Wang et al. 1998, Wei et al. 2010).

## Supplementary Material

XML Treatment for Deinostigma fasciculatum
